# Healthy for My Baby Research Protocol- a Randomized Controlled Trial Assessing a Preconception Intervention to Improve the Lifestyle of Overweight Women and Their Partners

**DOI:** 10.3389/fpubh.2021.670304

**Published:** 2021-08-03

**Authors:** Isabelle Hardy, Amanda Lloyd, Anne-Sophie Morisset, Felix Camirand Lemyre, Jean-Patrice Baillargeon, William D. Fraser

**Affiliations:** ^1^Department of Obstetrics and Gynecology, University of Sherbrooke and Centre de Recherche du Centre Hospitalier Universitaire de Sherbrooke (CRCHUS), Sherbrooke, QC, Canada; ^2^Institute of Biological, Environmental, and Rural Sciences, Aberystwyth University, Aberystwyth, United Kingdom; ^3^School of Nutrition, Faculty of Agricultural and Food Science, Laval University, Laval, QC, Canada; ^4^Department of Mathematics, University of Sherbrooke and CRCHUS, Sherbrooke, QC, Canada; ^5^Endocrine Division, Department of Medicine, University of Sherbrooke and CRCHUS, Sherbrooke, QC, Canada

**Keywords:** preconception care, pregnancy, overweight and obesity, lifestyle intervention, health technology, randomized controlled trial, biomarkers/urine, metabolomics

## Abstract

**Background:** Preconception lifestyle interventions appear promising to reduce pregnancy complications, prevent adult cardiometabolic diseases, and prevent childhood obesity. These interventions have almost exclusively been studied in populations of obese infertile women. The development of preconception lifestyle interventions targeting a broader population of overweight and obese women without a history infertility and their partners is needed.

**Methods:** This study is a multicenter open label parallel group randomized controlled trial. Sixty-eight non-infertile women with overweight or obesity in the preconception period and their partners will be recruited from the Sherbrooke and Quebec City regions. The couples will be randomized in a 1:1 ratio to receive the *Healthy for my Baby* intervention or standard care in the preconception period and pregnancy. Women and their partners will be invited to take part in this lifestyle intervention which includes motivational interviews and daily self-monitoring of lifestyle goals through a mobile phone application. The primary endpoint of this study is the diet quality of women during the preconception period, which will be evaluated using the C-HEI 2007 score at baseline, 2, 4- and 6-months following study enrolment. Women's dietary quality will also be evaluated through the measure of urinary biomarkers of habitual dietary intake at baseline and 2 months in preconception, and 24–26 weeks in pregnancy. Additional indicators of women's lifestyle as well as anthropometric measures will be documented in preconception and pregnancy. For the pregnancy period, the main secondary endpoint is the pattern of gestational weight gain. Pregnancy and neonatal complications will also be evaluated. For partners, diet quality, other lifestyle habits, and anthropometric measures will be documented in the preconception and pregnancy periods.

**Discussion:** This study will evaluate the effectiveness of a low-cost intervention designed to improve diet and other lifestyle characteristics of women in the preconception period who are overweight or obese. If the *Healthy for my Baby* intervention is efficacious regarding dietary measures, larger trials will be needed to evaluate the impact of this intervention on the rates of pregnancy complications, childhood obesity, and adult cardiometabolic disease.

**Clinical Trial Registration:**clinicaltrials.gov (NCT04242069).

## Introduction

### The Obesity Epidemic

Overweight and obesity are major risk factors for non-communicable diseases (1) and became pandemic worldwide at the end of the 20th century (2). In the early 2000's, the World Health Organization (WHO) recognized the importance of preventing obesity in order to improve the health of populations (3). Increasing physical activity and improving diet quality are major goals of the 2013 *Global Action Plan for the Prevention and control of Non-communicable Diseases* (4) and should help reduce the prevalence of overweight and obesity. Despite these efforts, global adult obesity rates have nearly tripled from 5 to 15% between 1975 and 2016. Obesity rates are alarmingly high in Canada and the United States where 30 and 38% of adults are obese, respectively (5). Most strikingly, childhood obesity rates have increased by more than 700% during this period, and continue to climb rapidly in Africa and South-East Asia (5). Given the inefficacy of current strategies aimed at preventing adult and childhood obesity, new intervention strategies are urgently needed.

### Lifestyle Interventions in the Preconception Period

In the effort to prevent obesity across the life course, interventions in the preconception period appear particularly promising (6). Helping women with overweight or obesity to improve their metabolic health in the preconception period has the potential to diminish pregnancy complications, to improve the long-term cardiometabolic health of these adults, and to prevent childhood obesity of their offspring.

Women with overweight or obesity who enter pregnancy are at increased risk of several complications including gestational diabetes mellitus (GDM), hypertensive disorders of pregnancy (HDP), fetal macrosomia, and delivery by cesarean section (7). These women are also more likely to gain excessive gestational weight (8), which is an independent risk factor for macrosomia and delivery by cesarean section (9). Several lifestyle intervention studies targeting women with overweight or obesity have been conducted in pregnancy, but they have failed to demonstrate a significant effect on excessive gestational weight gain or maternal and neonatal complications. The results of a recent individual patient data meta-analysis demonstrated that antenatal diet and physical activity interventions are associated with a modest reduction in both gestational weight gain and cesarean section rates, but do not have a significant impact on any other maternal or neonatal outcomes (10). Observational data from a Canadian cohort of over 200,000 pregnant women revealed that for each 10% decrease in maternal pre-pregnancy body mass index (BMI), pregnancy complication rates are lower by more than 10% (11). It is therefore plausible that a preconception intervention aimed at improving the lifestyle habits of women with overweight or obesity could help reduce pregnancy complications through direct impacts on metabolism, weight loss, and the prevention of excessive gestational weight gain. This hypothesis is further supported by evidence from post-bariatric surgery cohorts, which demonstrate a complete reversal of obesity-associated pregnancy complications after weight loss (12).

Intervening to improve the lifestyle habits of women with overweight or obesity and their partners in the preconception period could also prevent the increase in body weight associated with parenthood and improve the long-term metabolic health of adults. Indeed, women and men who become parents are more likely to gain weight and increase their BMI than their childless counterparts (13–15). Furthermore, parity is a risk factor for becoming overweight in non-smoking women (16), and the transition to overweight is partially predicted by excessive gestational weight gain (17). The preconception period thus appears as a promising intervention window to prevent adult overweight and obesity.

Lastly, lifestyle interventions targeting women with overweight or obesity and their partners in preconception could help prevent the transgenerational transmission of overweight and obesity. The transmission of an increased risk of overweight and obesity from parents to their children has been well-documented (18, 19), and several mechanisms have been proposed to explain this phenomenon including metabolic disturbances during gamete formation and fetal life, mitochondrial DNA, epigenetics, microbiota, non-coding RNAs in seminal fluid, and shared behaviors (20–22). The potential impacts on offspring health of a lifestyle intervention targeting couples with overweight or obesity in preconception are 2-fold. From a biological stance, allowing women with overweight or obesity to adopt healthy habits prior to pregnancy could restore a normal metabolic environment for the critical period of embryo programming. According to the theory of the Developmental Origin of Health and Disease (DOHaD), the restoration of a normal metabolic environment must already be in place in the early first trimester for the future child to be protected against metabolic disease through epigenetic changes (23). Healthy habits adopted prior to pregnancy could also prevent excessive gestational weight gain which is an important mediating factor for the risk of childhood overweight and obesity (24, 25). From a behavioral perspective, encouraging women and their partners to adopt healthy habits prior to pregnancy may increase the likelihood that children will be brought up in an environment where healthy eating, physical activity, sleep, and mental health are embodied (26, 27). In this regard, including male partners in preconception interventions is crucial, since the weight status and lifestyle habits of the fathers has been closely correlated with that of their child (28–30).

### The Dearth of Evidence on Preconception Interventions

Several preconception lifestyle interventions have been developed using telephone coaching, face-to-face coaching, motivational interviews, and information technology and are being evaluated in ongoing clinical trials (31–33). Only one randomized controlled trial with published results has evaluated the effectiveness of a lifestyle intervention targeting non-infertile women with overweight or obesity in preconception, and none have included women's partners (34–36). In the preconception arm of the RADIEL trial (37), 228 women at high risk of GDM (with a personal history of GDM or a BMI ≥ 30) were randomized to standard care or a lifestyle intervention consisting of advice on diet and physical activity provided through personalized in-person meetings every 3 months. This study did not show a difference in the incidence of GDM between the control and intervention groups. However, these negative results could at least partially be explained by the higher pregnancy rates amongst women with obesity in the intervention group (52% of pregnant women were obese in the intervention group compared with 33% in the control group), which rendered the groups imbalanced when considering pregnancy outcomes (38).

Given the high burden of overweight and obesity on public health and the promising potential of lifestyle interventions targeting couples with overweight or obesity in preconception, further research is needed to elaborate and evaluate such interventions. The aim of this paper is to report the rationale and methods for the *Healthy for my Baby* trial, a randomized controlled trial evaluating the effectiveness of an intervention combining in person contacts with an information technology (IT) support to improve the lifestyle habits of women with overweight or obesity in preconception. This protocol is written in alignment with the 2013 SPIRIT statement (39). The SPIRIT checklist for this clinical trial is provided in Supplementary File 1.

### Hypothesis

*Healthy for my Baby* is a novel behavioral intervention developed by our research team to support the adoption of healthy lifestyle habits for women with overweight or obesity, and their partners, in preconception and throughout pregnancy. Our hypothesis is that, compared with standard preconception and obstetrical care, this intervention will lead to:

- An improvement in the lifestyle habits of women and their partners in the preconception period and during pregnancy.
- A reduction in women's weight in the interval from enrolment to either (a) occurrence of a pregnancy or (b) in the absence of pregnancy, 6 months of follow-up.

Among women who achieve pregnancy, we also aim to generate preliminary data on the effect of the intervention on the proportion of women who adhere to gestational weight gain guidelines as well as on rates of complications in pregnancy and the neonatal period.

## Methods and Analysis

### Objectives

In accordance with the Obesity Related Behavioral Intervention Trials Consortium model for the development and evaluation of interventions, the primary aim of this study is to determine the impact of the intervention on disease risk factors (40). This trial will be conducted in two phases. The preconception phase will evaluate the impact of the intervention on preconception risk factors for pregnancy, neonatal, and metabolic complications, which include lifestyle habits and anthropometric measures. These outcomes will provide an insight on the mechanisms through which the intervention might improve clinical outcomes. The pregnancy phase will evaluate whether the intervention has a sustained impact on lifestyle habits, and will allow us to obtain preliminary estimates of potential impacts on clinical maternal and neonatal outcomes.

The primary outcome of the trial is the diet quality of women, assessed with the Canadian Healthy Eating Index (C-HEI) during the preconception period, as it is sensitive to change and has been identified as an independent predictor of gestational diabetes, hypertensive disorders of pregnancy, birthweight, and neonatal fat mass (41–44). This outcome is also associated with parallel changes in other behaviors, such as physical activity, sleep, well-being and environment, which are however less sensitive to change over a relatively short period. Since this trial is the initial evaluation step in the evaluation of a novel intervention, we have chosen to base the power of the trial on this disease risk factor, with all other outcomes being secondary at this point.

We set the maximum duration of preconception follow-up at 6 months as fertility rates tend to plateau or decline after 6 months of attempting to achieve pregnancy (45).

**For the Preconception Period:**

- The primary objective of this trial is to evaluate the impact of the intervention on the diet quality of women measured with the Canadian Healthy Eating Index at 2, 4, and 6 months of follow-up.
- The secondary objectives of the preconception period are to evaluate the impact of the intervention on:
  - Urinary metabolomic indicators of women's dietary exposure at 2 months follow-up,
  - The diet quality of male partners at 2, 4, and 6 months follow-up,
  - The other lifestyle habits of women and their partners at 3 and 6 months (physical activity, sleep quality, anxious and depressive symptoms, and quality of life),
  - The anthropometric measures of women and their partners at 3 and 6 months (weight, waist circumference, and body fat percentage), and
  - The proportion of women and partners with a weight loss of at least 5% body weight at 3 and 6 months.

After completion of the primary outcome assessment, women and their partners who have achieved a pregnancy within 12 months following enrolment will be followed until the end of the pregnancy. Although, there is a potential for unbalanced study groups if the intervention has an impact on fertility, this exploratory follow-up phase will provide preliminary data on the impact of the intervention on lifestyle and clinical outcomes in pregnancy.

**For Pregnancy:**

- The main secondary objective of this phase is to explore the impact of the intervention on the proportion of women whose gestational weight gain conforms to the guidelines of the *National Academy of Medicine* recommendations (46).
- The other secondary objectives of the pregnancy period are to explore the impact of the intervention on:
  - The trajectory of dietary and lifestyle habits of women and their partners in the first, second, and third trimesters,
  - Women's urinary dietary exposure metabolomic profile at 24–26 weeks of pregnancy,
  - The change in the anthropometric measures (weight, body fat percentage, and waist circumference) of partners between the first and third trimesters,
  - The proportion of partners with overweight or obesity at study enrolment who have a weight loss of at least 5% body weight in the first, second, and third trimesters,
  - Perinatal morbidity indicators (gestational diabetes, hypertensive disorders of pregnancy, gestational age at delivery, delivery mode, birthweight, and neonatal hypoglycemia),
  - Fertility outcomes (viable pregnancy rate, spontaneous abortion rate, and live birth rate).

Women's diet quality will also be assessed based on metabolomic analysis of urinary samples. We will establish a profile of dietary exposure biomarkers, which will be used to compile a metabolite derived diet quality score (47, 48). This exploratory analysis will provide important data to support its use in a future larger multicenter trial.

**Exploratory Objectives:**

- To evaluate the correlation between women's C-HEI score and the metabolite derived diet quality score in preconception and pregnancy.
- To explore untargeted metabolomic phenotypes within the study population to find associations between metabolism and eating patterns during pregnancy.

### Trial Design and Setting

This study is a multicenter open label parallel group randomized controlled trial coordinated at the Research Centre of the Centre hospitalier universitaire de Sherbrooke (CR-CHUS) in Sherbrooke, Quebec, Canada. The Province of Quebec has a population of 8.5 million. In 2014 overweight and obesity rates were 27 and 15% for adult women and 41 and 18% for adult men, respectively (49). Participants will be recruited from the Sherbrooke and Quebec City regions. The obstetrical care of women from Sherbrooke and Quebec City is provided by general practitioners, midwives, and obstetricians in outpatient clinics. The CHUS is a regional tertiary care center where ~2,800 deliveries are performed annually. The CHU de Québec Université Laval is a regional tertiary center where ~7,500 deliveries are performed annually at two sites, the Centre Hospitalier de l'Université Laval and the Hôpital Saint-François d'Assise.

An explanatory design has been chosen to rigorously evaluate the potential benefits of the *Healthy for my Baby* intervention (50). Masking of the research team and participants to the intervention will not be possible given the nature of the study. Couples will be randomized to the intervention or control group in a 1:1 ratio. The randomization list was generated independently by the CRCHUS' biostatistics service using a computerized unstratified blocked randomization with blocks of random sizes 2–6. The group allocation sequence was sent directly to the mobile application programming team who input it into the mobile application software. The mobile application website is hosted on the PIERCE server, a secured research platform developed by the local CRED medical informatics working group. Once participants have provided written consent, completed the trial enrollment form, and completed baseline assessment, their mobile application profile will be created by the research assistant which will trigger randomization to a treatment group. Participants will receive the result of their group allocation by email with a link to install the appropriate version of the mobile application.

### Eligibility Criteria and Recruitment

Women in the preconception period will be considered for enrollment if they meet the following eligibility criteria. Inclusion criteria: (1) Age 18 to 40 years old, (2) BMI ≥ 25 kg/m^2^, (3) the participant intends to conceive within 12 months of trial enrollment, (4) access to a smart phone. While participation of the woman's partner will be strongly encouraged, it is not mandatory for inclusion in the study. Women attempting to conceive by insemination without a history of infertility and same sex partners are also eligible for trial inclusion. Exclusion criteria applicable to all participants: (1) anticipate move to another region, (2) insufficient knowledge of French or English, (3) personal history of infertility. Exclusion criteria applicable to women only: (4) type 1 or 2 diabetes mellitus, (5) prior bariatric surgery, (6) active eating disorder established by clinical diagnosis, (7) medical contraindication to pregnancy, (8) medical contraindication to physical activity, (9) known or anticipated disease or surgery likely to cause an important weight loss. For exclusion criteria 7, 8, and 9, the physician overseeing the day-to-day operations of the study (IH) will individually review the participant's medical history to determine if they present one of these conditions. If a multiple pregnancy occurs, study follow-up will be stopped to limit potential confounders.

Recruitment of eligible volunteers from the Sherbrooke and Quebec City region began in June 2021 and should be completed within 24 months. Potential participants will be approached by their care provider in general practice and obstetrics and gynecology clinics, or at the emergency department after a spontaneous first trimester abortion. Potential participants will also be approached through advertising on social media and in community drugstores. Postpartum women according to hospital archives will be sent mail invitations to participate in the study at the time that they are considering another pregnancy. If these strategies are ineffective, a list of women who are 6 months postpartum will be obtained from the hospital archives and these women will be sent reverse contact authorization forms through the mail. Women who do not return the form within a month will be considered to have consented to be contacted by the research team.

### Interventions

#### Intervention Arm

Participants randomized to the intervention group will receive the *Healthy for my Baby* intervention. This intervention is based on the Control Theory, according to which behavior can be regulated by engaging oneself in an active process centered on establishing and following-up specific goals (51). Couples will initiate the preconception intervention by participating in a 60-min motivational interview (MI) session on healthy lifestyle habits (52). At the end of this session, each member of the couple will have set specific SMART lifestyle goals (53) in at least two of five key areas: nutrition, physical activity, sleep, well-being (stress, anxiety), and environment (tobacco use, drug use, alcohol consumption, or other toxic substances). Lifestyle goals will be tailored to each participant based on the priorities verbalized in the motivational interview with the aim of improving compliance with Canadian guidelines for diet, physical activity, sleep, and consumption of alcohol (54, 55). Participants will then have access to a mobile phone application designed by our research team that will help them achieve three goals in different dimensions at a time through daily self-monitoring (Supplementary Figure 1). Self-monitoring has been shown in a meta-regression analysis to be the most effective behavior change strategy to improve diet and physical activity (56). In addition to allowing participants to track their lifestyle goals, the mobile application includes references on healthy habits for preconception and pregnancy, a research visit calendar, a fertility tracker, links to videos on different aspects of parenthood, and a platform to contact the research team.

One month after enrolment, couples will participate in a second 30-min MI to reevaluate their objectives and help resolve potential obstacles. They will also be invited to set new SMART goals in three of the five key areas. Participants will continue to have access to the mobile application for the remainder of the preconception follow-up. The research team will have access to the information entered in the mobile application and will use this data to determine if the intensity of the intervention needs to be increased. Participants who successfully reach their lifestyle goals will not have any further in-person meetings in the preconception period. Those who fail to meet their lifestyle goals will be contacted for further support and will be invited to participate to a maximum of two additional monthly motivational interview sessions by videoconference. Participants will have the option to track their weight in the mobile application and will be contacted for further support if weight gain is reported. Several reminders have been embedded in the application to improve adherence to the intervention. The research team will also be able to detect participants who are not using the application on a regular basis to contact them for further support.

If the woman becomes pregnant, the application will be put in pregnancy mode and participants will be invited to engage in two additional 30-min MI sessions at 6–8 and 10–12 weeks of gestation. The aim of these additional encounters is both to intensify the intervention in the critical period of early pregnancy and to tailor the lifestyle goals to the symptoms of pregnancy. Participants will keep using the mobile application for self-monitoring throughout pregnancy and up to three additional monthly videoconference meetings will be planned for those requiring further support according to data derived from the application.

#### Control Arm

Participants randomized to the control group will receive standard advice on healthy lifestyle habits as provided by their usual care provider. Usual care in preconception is the same as that of healthy adults and does not specifically involve access to lifestyle interventions. In the Estrie region, which includes the Sherbrooke area, individuals with obesity, or overweight and a cardiometabolic diagnosis, have access to a free-of-charge program at local health centers, which includes group sessions with a limited individual nutritional intervention. However, these services are neither integrated nor targeted at preconception care and are rarely used by these couples. Participation in these sessions, or in any other lifestyle programs or consultations with a lifestyle professional will be documented. In addition to routine obstetrical care, women with GDM are typically followed by a nutritionist, and those who present comorbidities such as hypertension or morbid obesity may be followed by a nutritionist. Participants in this group will have access to a simplified version of the mobile application containing only the research visit calendar, the fertility tracker, videos on parenthood, and the platform to contact the research team. This version of the application does not contain any information on healthy lifestyle habits.

Participants from both study groups will be allowed to participate in the existing local *Taking Care of your Health* educational program if they are eligible or to consult any health care professional if clinically required. However, they will be asked not to voluntarily begin a similarly intense lifestyle program during the trial.

### Variables and Data Collection

Participants' medical history and socioeconomic status will be evaluated at baseline. Women and their partner's diet quality will be measured with 24-h dietary recalls at baseline, 2, 4, and 6 months in preconception and at 6–8 weeks, 24–26 weeks, and 32–34 weeks in pregnancy. Women will provide urinary samples for the metabolomic analysis at baseline and 2 months in preconception and at 24–26 weeks in pregnancy. Women and their partner's other lifestyle indicators (physical activity, sleep, anxious, and depressive symptoms, quality of life, and attitude toward behavior change), and anthropometric outcomes will be evaluated at baseline, 3 and 6 months in preconception and every trimester in pregnancy. Information on pregnancy and neonatal outcomes will be collected from participants' medical records at the end of the study. The study variables, measuring tools, and the timing of data collection have been summarized in Table 1.

**Table 1:** Summary of study variables.

		**Timing of data collection in preconception (months)**					**Timing of data collection in pregnancy (weeks of amenorrhea)**			
**Variables**	**Measuring Tool**	**0**	**2**	**3**	**4**	**6**	**6-8**	**24-26**	**32-34**	**End of study**
Medical history	Homemade questionnaire	•								
Socioeconomic status	Homemade questionnaire	•								
**Lifestyle habits**										
Diet quality	C-HEI 2007 score measured with the R24W	•	•		•	•	•	•	•	
Diet quality	Urine dietary exposure biomarker profile	♀	♀					♀		
Physical activity	IPAQ	•		•		•	•	•	•	
Active minutes and step count	7-day Fitbit recording	•		•		•	•	•	•	
Sleep quality	PSQI	•		•		•	•	•	•	
Anxious and depressive symptoms	HADS	•		•		•	•	•	•	
Quality of life	SF-12	•		•		•	•	•	•	
Attitude toward behavior change	Homemade questionnaire	•		•		•	•	•	•	
**Anthropometric measures**										
Height	Stadiometer	•								
Weight	Calibrated scale	•		•		•	•	•	•	
Body fat percentage	Foot to foot bioimpedance	•		•		•	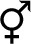	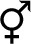	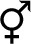	
Waist circumference	Measuring tape	•		•		•	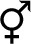	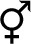	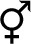	
**Pregnancy outcomes**										
Last weight before delivery	Patient medical record									♀
Gestational diabetes	Patient medical record									♀
Hypertensive disorder of pregnancy	Patient medical record									♀
Gestational age at delivery	Patient medical record									♀
Delivery mode	Patient medical record									♀
**Neonatal outcomes**										
Birthweight	Patient medical record									♀
Neonatal hypoglycemia	Patient medical record									♀
**Fertility outcomes**										
Clinical pregnancy rate	Patient medical record									♀
Spontaneous abortion rate	Patient medical record									♀
Live birth rate	Patient medical record									♀

*•: Evaluated for both partners. ♀ and 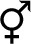: Evaluated for the woman attempting pregnancy or partners only, respectively*.

#### Lifestyle Habits

##### Diet Quality

Diet quality will be measured using the *Canadian Healthy Eating Index* 2007 (C-HEI 2007) which is a 100-point score adapted from the American HEI-2005 to measure adherence to the 2007 Canadian Food Guide (57, 58). The C-HEI 2007 score will be measured at baseline, 2, 4, and 6 months in preconception and each trimester in pregnancy with the *R24W*, an online 24-h dietary recall tool available in French and English, and validated for both pregnant and non-pregnant individuals of the French-Canadian population (59, 60). Three dietary recalls will be used to compile the C-HEI score at each time point, two recalls of weekdays and one recall of a weekend day. On the day the 24-h recall is to be completed, an e-mail will be sent to the participant. If the participant fails to complete the R24W, subsequent e-mails will be sent, within 3 weeks (or within the trimester), until three R24W are completed. Detailed reports of energy, macro and micronutrient, are automatically derived from the recall and will also be compiled and analyzed. Furthermore, a new diet quality index reflecting the 2019 Canadian Food Guide might become available to assess diet quality and will be reported if applicable.

Women's urinary dietary exposure biomarker profiles will be analyzed as a secondary assessment of diet quality. A crossover randomized controlled trial published in 2017 demonstrated that urinary metabolic profiling is a precise tool to evaluate dietary intake of the preceding 72 h, that it is sensitive to change, and that it correlates well with the DASH dietary index (61). Participants will collect urine samples on three random days over 1 week at the designated time frame. These spot samples will be pooled after normalizing using refractive index measurement to guide dilution. Around 40 metabolites will be targeted for the quantitative measurement of dietary intake biomarker concentrations. The concentration of dietary intake biomarkers will be measured using Multiple Reaction Monitoring (MRM) methods on a Triple Quadruple LC-MS/MS instrument (47). Each metabolite can be associated with the consumption of a specific food. These foods will then be grouped together into Healthy Eating Index categories to compute a relative dietary quality score (48).

##### Physical Activity

Physical activity behavior will be assessed at each research visit using the *International Physical Activity Questionnaires* (IPAQ) (62).

##### Active Minutes and Step Count

The minutes of moderate to intense physical activity and the daily step count will be measured after each research visit using a 7-day Fitbit recording (Flex 2 model). The use of the Fitbit monitor to measure these variables has been validated both in the general (63, 64) and pregnant population (65).

##### Sleep Quality

Sleep quality will be evaluated after each research visit using the *Pittsburgh Sleep Quality Index* (PSQI) (66).

##### Anxious and Depressive Symptoms

The intensity of anxious and depressive symptoms will be evaluated at each research visit with the *Hospital Anxiety and Depression Scale* (HADS). Both the anxiety (HADS-A) and depression (HADS-D) dimensions of this scale have been extensively validated in the general (67) and gynecological population (68). This questionnaire can also be utilized as a screening tool for anxious and depressive disorders with a cut-off value of 8 or greater on the HADS-A or HADS-D subscales (67, 68).

##### Quality of Life

Quality of life will be evaluated at each visit using the SF-12, a shortened version of the SF-36 which evaluates both physical and mental health and has been validated in the gynecological population (69).

##### Attitude Toward Behavior Change

The readiness, conviction, and confidence of study participants to change their diet and physical activity will be measured using a 22-item questionnaire. This questionnaire was designed for previous studies (70, 71) at our center and is based on Prochaska and DiClemente's transtheoretical model of change (72).

##### Anthropometric Measures

All anthropometric measures will be performed by a research assistant who has been trained in these measures based on WHO standards (73).

#### Pregnancy Outcomes

##### Total Weight Gain in Pregnancy

Total weight gain in pregnancy will be calculated by subtracting the woman's weight at the last attended preconception visit from the last weight recorded before delivery. The last weight recorded before delivery will be obtained from the patient medical record. Trimester specific weight gain velocity will also be calculated based on the weight measured at the research visits. Trimester-specific gestational weight gain will also be computed using previously described methodology (74). Both total and trimester specific weight gain will be compared to the NAM recommendations (46).

##### Pregnancy Complications

The diagnoses of gestational diabetes or hypertensive disorders of pregnancy, gestational age at delivery, and delivery mode will be compiled from women's medical records based on *International Classification of Diseases-Tenth Revision* codes.

#### Neonatal Outcomes

The birthweight, and the presence of hypoglycemia will be compiled from the newborn medical records.

#### Fertility Outcomes

The clinical pregnancy rate, spontaneous abortion rate, and the live birth rate will be compiled from the patient medical records.

### Participant Timeline

**Figure 1:**
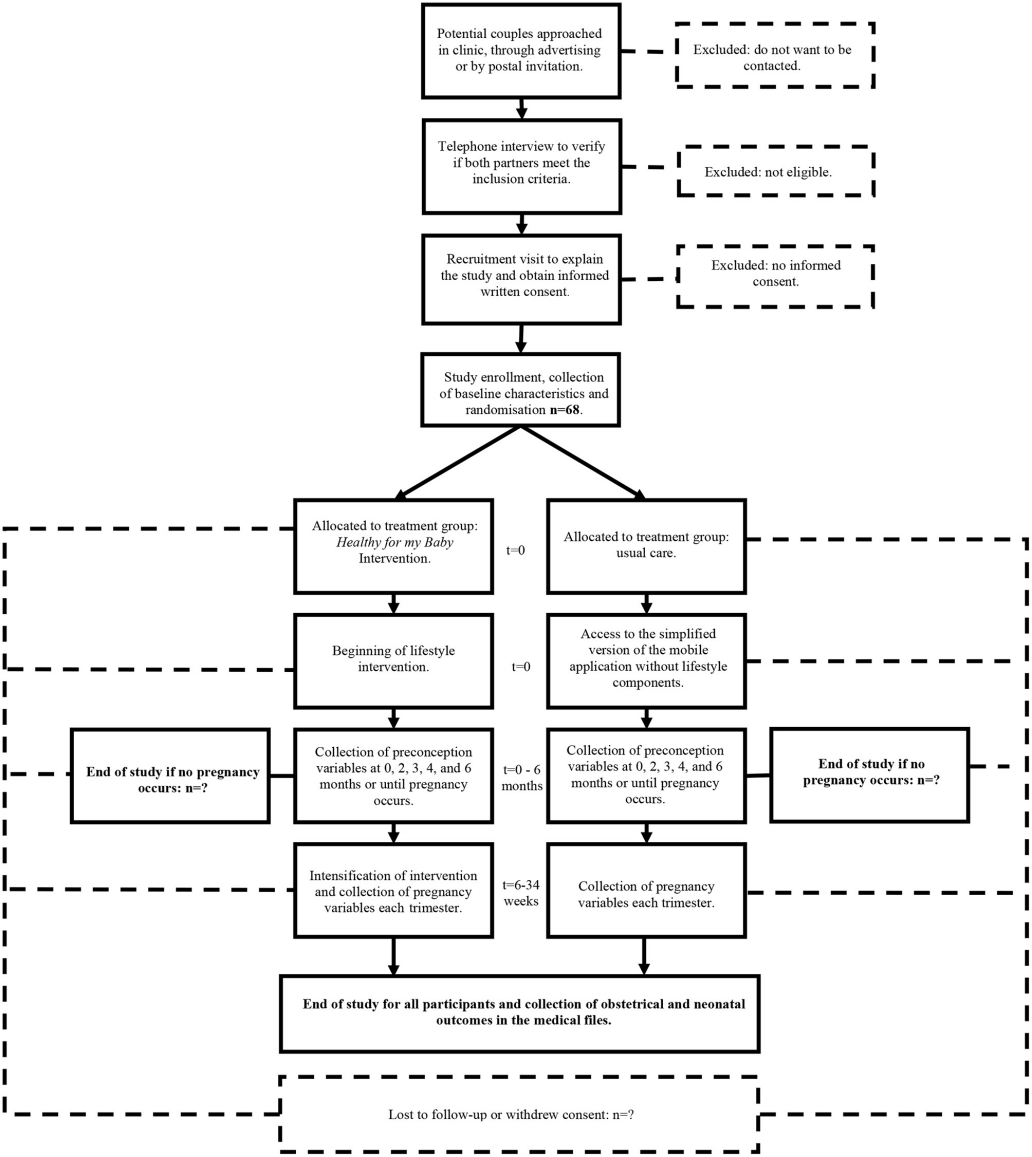
Participant timeline.

The participant timeline has been summarized in Figure 1.

### Sample Size Calculation and Analysis Plan

#### Sample Size Calculation

Since no minimally clinically important effect size has been defined for the HEI score, our effect size has been estimated as a plausible effect of the intervention based on the preliminary results of the *Obesity Fertility* trial (71), which demonstrated a 12.5 ± 13.0 points increase in the average HEI score of women with obesity and infertility after a 6-month lifestyle intervention (unpublished data). An increase of 12 points in the average HEI score was also observed in breast cancer survivors with obesity who received a 6-month dietetic intervention (75). To detect a 10% difference in the average HEI score between groups with a 13-point standard deviation, an alpha value of 5% and 80% power, we will need to recruit 27 women per group (76). To account for a 20% attrition rate (77), a total of 68 women and their partners will be recruited.

#### Statistical Analysis Plan

Study results will be analyzed at the end of the trial following intent-to-treat principles. Participants' baseline characteristics will be reported to assess group comparability at baseline in preconception and at the beginning of pregnancy. A statistical significance threshold of 5% will be used for all analyses. Statistical analyses will be performed using the Statistical Package for the Social Sciences (SPSS version 26) and R (Version 4.0.2) software. No interim analysis will be performed.

#### Primary Outcome Analysis

Our primary endpoint is the difference in the evolution of the HEI score between study groups in time, which will be assessed using a mixed linear model of the HEI score integrating the effects of time as a continuous variable, study group, and the interaction between the study group and time. This statistical model has been chosen because it will allow us to account for (1) the different lengths of preconception follow-up that will arise as a result of women becoming pregnant throughout the study; and (2) variable timing and number of the *R24W* questionnaires performed by the participants (due to non-adherence to the protocol) (78). The average HEI score in each group at 2, 4, and 6 months will also be compared using standard mixed-model repeated measures ANOVA with *post-hoc* Student's *t*-tests with Bonferroni correction for multiple comparisons. As a secondary analysis, Kaplan-Meier survival curves of women who have increased their HEI score by 10 points or more will be produced, with censoring for dropout or occurrence of pregnancy.

#### Secondary Outcome Analysis

For the main secondary outcome for the pregnancy phase of the trial, we will use a Chi-squared test to compare the proportion of women who have an adequate gestational weight gain in each group.

For the targeted analysis of urine biomarkers, the concentration of around 40 metabolites will be compared between baseline, 2 months in preconception and 24–26 weeks of pregnancy using repeated measures ANOVA or Friedman test with a Bonferroni adjustment for multiple comparisons. The correlation between the metabolite derived diet quality score and the C-HEI score will be evaluated using Pearson's correlation.

Although our sample size is fairly small, an exploratory multivariate analysis of non-targeted metabolome profiles will be realized using both unsupervised ‘natural clustering’ analysis and Supervised Random Forest classification.

For the other secondary outcomes, continuous variables will be assessed using Student's *t*-tests or Mann-Whitney tests as appropriate. Dichotomic variables will be analyzed using Chi-squared tests or Fisher's exact test as appropriate.

For participants who achieve a pregnancy prior to the 2-month food recall and/or 3-month preconception visit, lifestyle and anthropometric outcomes at the first pregnancy visit will be used to complete the preconception analysis. Women will be able to signal the occurrence of a pregnancy through the mobile application as soon as a period is missed. The application software will immediately notify the research team which will allow completion of data collection prior to the occurrence of significant pregnancy symptoms such as hyperemesis.

### Retention Strategies

Participant retention is critical in all randomized controlled trials and may be even more important in open-label trials where differential attrition of study participants can occur and severely compromise the study results. Several strategies derived from a meta-analysis and expert recommendations have been put in place in order to improve the retention of study participants in this trial (79, 80) including thorough counseling of potential participants before enrolment, creation of a strong study brand, use of technology to ease the burden of participation (research calendar embedded in the mobile application, online dietary recalls with the R24W, email recalls), and flexible hours for study visits. Complementary healthy snacks and drinks will be offered at each study visit and parking fees will be assumed by the research team. After each research visit, participants will gain access to a new video through the mobile application, which will serve as a non-financial participation incentive. These videos have been created in collaboration with a local research team and provide scientifically accurate information on various aspects of parenthood. A fertility planner has been integrated in the mobile application as another non-financial participation incentive. Monetary compensations for time or travel will not be provided for this trial.

### Safety Monitoring

In order to document the safety of the *Healthy for my Baby* intervention, we have chosen to follow expert recommendations (81, 82) and implement a comprehensive adverse event monitoring strategy. Monitored adverse events include subjective anxious or depressive symptoms, a score of 8 or higher on the anxiety or depression subscale of the HADS questionnaire (68), physical trauma related to an increase in physical activity, placental abruption secondary to a trauma incurred in the context of an increase in physical activity, excessive weight loss on the part of the woman defined as the loss of more than 1 kg per week, intrauterine growth restriction associated with excessive maternal weight loss. These adverse events were defined based on plausible consequences of the intervention because no specific adverse events have been reported in association with lifestyle weight loss interventions (83). All adverse events will be reported to the DSMB, which will meet every 6 months or more often if needed, to evaluate the nature and rate of adverse events and determine their association with the intervention. If a severe adverse event occurs (requiring hospitalization, causing long-term morbidity, or death) the DSMB and local ethics board will be immediately notified to evaluate the need for trial interruption. No study termination criteria have been defined a priori.

## Discussion

This innovative study will be the first to document the effectiveness of an intervention combining motivational interviews and IT support to improve the lifestyle habits of women with overweight or obesity without a history of infertility in the preconception period (34, 35). Furthermore, it will be the first study to include male or female partners in the evaluation of a preconception intervention (36). In addition to documenting the impact of the intervention on the lifestyle habits and anthropometric indices of partners, this analysis will provide insight on the impact of sex and gender in the response to interventions, and will allow us to explore whether the participation of both members of the couple is of added benefit for the achievement of lifestyle goals by each participant.

The body of evidence produced in this trial will be useful to guide future research endeavors on obesity prevention in preconception and pregnancy. This study will document the impact of the intervention on nutritional behaviors in preconception and will provide evidence regarding the plausibility of an effect of the intervention on health outcomes in the preconception period and pregnancy. If the intervention produces positive dietary changes which are sustained through the preconception period and pregnancy, a larger trial will be warranted to assess the effects of the intervention on clinical outcomes of pregnancy.

The use of urinary metabolomic profiling will also allow us to explore possible associations between epidemiological measures of diet and biological (metabolomic) indictors that may reflect dietary changes produced by the intervention. This profiling appears particularly promising as it is an objective means of evaluating dietary intake and is not subject to recall bias. If the metabolite derived diet quality score can be correlated with the C-HEI both in preconception and pregnancy, urinary dietary exposure biomarkers could be used as the sole measure of dietary intake and quality in future studies.

We will also document several measurements of the feasibility of targeting couples with no history of subfertility in the preconception period including recruitment rates, attrition rates, effective recruitment and retention strategies, and pregnancy rates within a 12-month preconception period. No similar study has been registered on the platforms clinicaltrials.gov or on the International Clinical Trials Registry Platform.

The methodology of this trial presents several strengths. Firstly, our intervention was designed to be in line with both the UK Medical Research Council statement on the development and evaluation of complex interventions, and the Consort statement for the reporting of non-pharmacologic interventions (84, 85). Indeed, our intervention is based on a recognized behavior change model, specific intervention targets have been outlined, and the behavior change techniques at play have been well-described. These elements will improve the likelihood of intervention success and allow replication of the intervention if it is proven to be effective. Secondly, we have chosen to document the effectiveness of the intervention on metabolic risk factors in the preconception period prior to conducting a large clinical trial with sufficient power to detect differences in clinical outcomes. This first step in evaluating a complex behavioral intervention is in line with the Orbit recommendations (40) and will avoid a waste of resources if the intervention appears to be ineffective. This will also avoid the limitations in outcome assessment found in the RADIEL trial (38), where the measure of the primary outcome in pregnancy, when randomization took place in preconception, severely compromised the validity of the results. Thirdly, the choice of an explanatory randomized controlled trial design will provide good internal validity to document the effects of the intervention. Lastly, our methodology includes a detailed and systematic approach to recruitment, retention, and adverse event monitoring which will all contribute to the validity and success of the study.

This study presents several limitations. First, our choice of diet quality in preconception as our primary endpoint, which is an intermediate variable for clinical outcomes, will limit the direct relevance of this study for clinical practice. Diet quality in preconception is also controversial, as two large cohort studies have reported that adhering to a combination of healthy habits including high diet quality, physical activity, being a non-smoker, low stress, and a normal BMI is more protective against the occurrence of GDM than any one of these factors alone (86, 87). However, for the purposes of sample size calculation, a single primary outcome had to be chosen and diet quality appears as the most predictive of pregnancy outcomes and is more sensitive to change (41–44, 58). Second, given the nature of the intervention, the masking of participants and the project coordination team to the study group will not be possible. Diet quality will be measured through an online 24-h dietary recall and urine samples, and the other lifestyle habits scores will be collected with the use of self-reported questionnaires to limit social desirability. Lastly, the strict selection of study participants could induce a selection bias and will limit the external validity of this trial. To limit this potential bias, we will use various recruitment methods to contact participants from diverse socioeconomic and cultural backgrounds and the baseline characteristics of participants in each group will be reported with our results.

We report the rationale and methods of an innovative randomized controlled trial evaluating the effectiveness of a low-cost intervention to support adoption of healthy lifestyle habits for women with overweight or obesity in the preconception period. This study will provide useful information on the feasibility of preconception trials, and on the potential of preconception interventions to prevent complications of overweight and obesity. This study will also help establish whether urinary metabolomic profiling can be used as a reliable and objective measure of diet quality in preconception and pregnancy. If the *Healthy for my Baby* intervention significantly improves lifestyle scores in women with overweight or obesity during preconception, larger trials will be needed to directly evaluate the impact of this intervention on clinical outcomes. Ultimately, if proven effective, this intervention could be integrated in regular clinical practice to help improve the health of couples, pregnant women and their babies. Future large-scale implementation of this intervention in diverse clinical settings will be facilitated by its low cost, ease of use and accessibility.

## Ethics and Dissemination

This study has been designed to follow the principles of the Canadian Tri-Agency Framework for the Responsible Conduct of Research (88). The approbations of the CHUS and CHU de Québec Université Laval Ethics Committees have been obtained prior to the start of recruitment. Any amendment to this protocol will be communicated to the Ethics Committees and updated on clinicaltrials.gov. All participants will be informed of the benefits and risks of study participation by a member of the research team or a research assistant and provide written informed consent before enrollment. Participants will be able to withdraw from the study at any point without consequences.

Urine samples will be anonymized and identified with the participant's study ID prior to being shipped to Professor John Draper's laboratory at Aberystwyth University. Urine samples will be destroyed after analysis.

Participant data will be de-nominalized and coded in a secured database. The coding key will only be available to the research team in a password-protected file. Study data will be stored for 10 years on a secured server and then destroyed. Study data will not be made publicly available and will only be accessible to the research team.

Study results will be disseminated through presentations at provincial, national, and international conferences and publications in peer-reviewed journals. The results will also be made available to the public through publication on clinicaltrials.gov and partnerships with patient advocacy groups. Presented and published results will not allow the identification of study participants.

## Author Contributions

IH wrote this manuscript and drafted the tables and figures. WF provided expertise on clinical trial design, complex interventions, maternal fetal medicine, adverse event monitoring, and statistical analysis. J-PB provided expertise in preconception lifestyle interventions, choice of relevant measuring tools, obesity medicine, and statistical analysis. AL provided expertise on urinary metabolomic profiling methods and on the analysis of urinary biomarker concentrations. A-SM provided expertise on the tools, timing, and analysis of nutritional outcomes. FC overlooked the statistical analysis plan. All authors read, edited, approved the final manuscript, and contributed significantly to the design of this clinical trial.

## Conflict of Interest

The authors declare that the research was conducted in the absence of any commercial or financial relationships that could be construed as a potential conflict of interest.

## Publisher's Note

All claims expressed in this article are solely those of the authors and do not necessarily represent those of their affiliated organizations, or those of the publisher, the editors and the reviewers. Any product that may be evaluated in this article, or claim that may be made by its manufacturer, is not guaranteed or endorsed by the publisher.
