# Supplementary material for: Healthy for My Baby Research Protocol- a Randomized Controlled Trial Assessing a Preconception Intervention to Improve the Lifestyle of Overweight Women and Their Partners
Source: Front Public Health. 2021 Aug 3;9:670304. doi: 10.3389/fpubh.2021.670304 (PMC8369366; doi:10.3389/fpubh.2021.670304)

Supplementary Figure 1- Overview of the mobile application

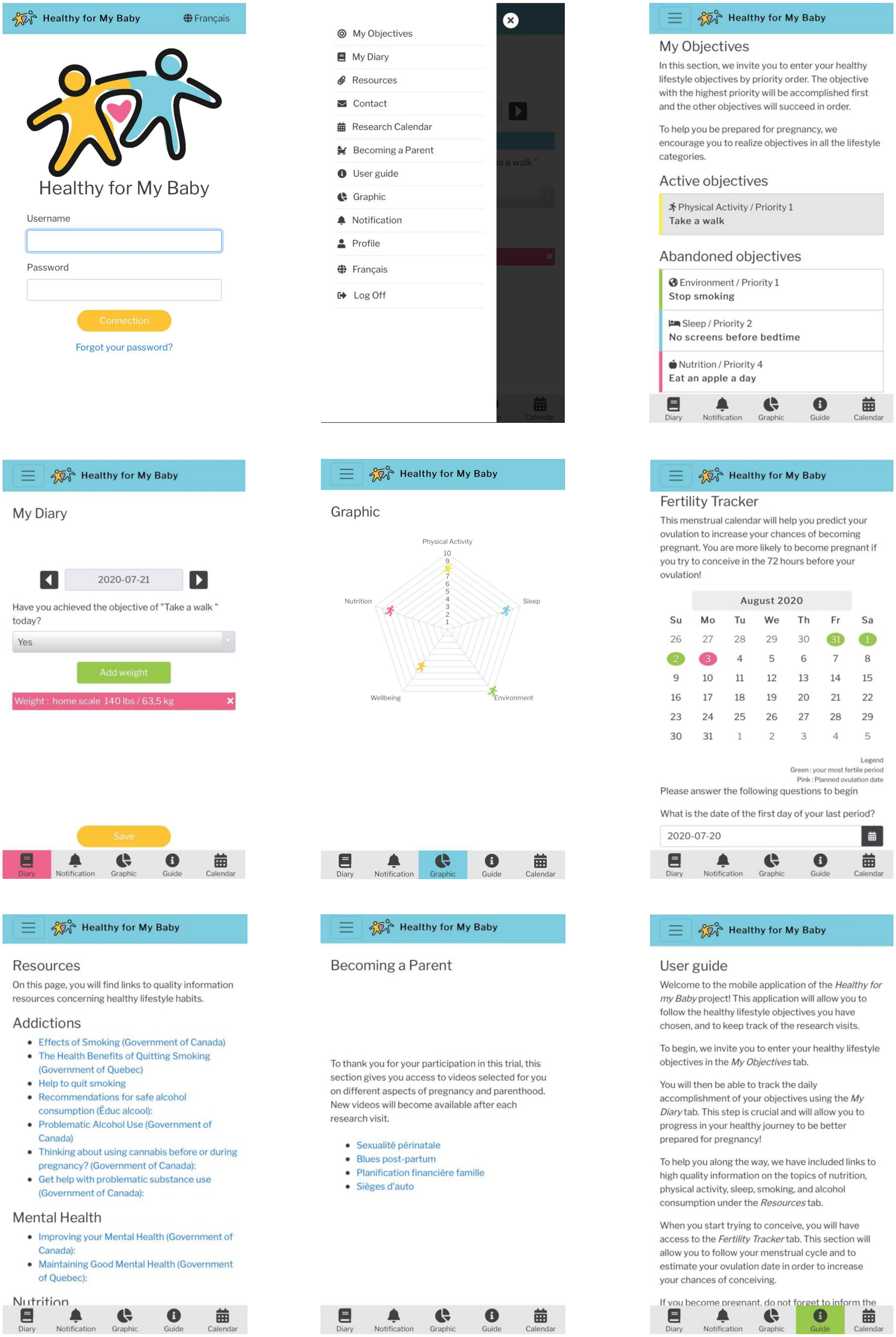

Supplement: Supplementary file 2 [file Image_1.PDF]
